# Convolutional block attention gate-based Unet framework for microaneurysm segmentation using retinal fundus images

**DOI:** 10.1186/s12880-025-01625-0

**Published:** 2025-03-10

**Authors:** C. B. Vanaja, P. Prakasam

**Affiliations:** School of Electronics Engineering, Vellore Institute of Technology, Vellore, India

**Keywords:** Diabetic retinopathy, Microaneurysm, Deep learning, Attention mechanism, Attention gate

## Abstract

**Background:**

Diabetic retinopathy is a major cause of vision loss worldwide. This emphasizes the need for early identification and treatment to reduce blindness in a significant proportion of individuals. Microaneurysms, extremely small, circular red spots that appear in retinal fundus images, are one of the very first indications of diabetic retinopathy. Due to their small size and weak nature, microaneurysms are tough to identify manually. However, because of the complex background and varied lighting factors, it is challenging to recognize microaneurysms in fundus images automatically.

**Methods:**

To address the aforementioned issues, a unique approach for MA segmentation is proposed based on the CBAM-AG U-Net model, which incorporates Convolutional Block Attention Module (CBAM) and Attention Gate (AG) processes into the U-Net architecture to boost the extraction of features and segmentation accuracy. The proposed architecture takes advantage of the U-Net’s encoder-decoder structure, which allows for perfect segmentation by gathering both high- and low-level information. The addition of CBAM introduces channel and spatial attention mechanisms, allowing the network to concentrate on the most useful elements while reducing the less relevant ones. Furthermore, the AGs enhance this process by selecting and displaying significant locations in the feature maps, which improves a model’s capability to identify and segment the MAs.

**Results:**

The CBAM-AG-UNet model is trained on the IDRiD dataset. It achieved an Intersection over Union (IoU) of 0.758, a Dice Coefficient of 0.865, and an AUC-ROC of 0.996, outperforming existing approaches in segmentation accuracy. These findings illustrate the model’s ability to effectively segment the MAs, which is critical for the timely detection and treatment of DR.

**Conclusion:**

The proposed deep learning-based technique for automatic segmentation of micro-aneurysms in fundus photographs produces promising results for improving DR diagnosis and treatment. Furthermore, our method has the potential to simplify the process of delivering immediate and precise diagnoses.

## Introduction

Diabetes is the primary cause of vision impairment and blindness. One common ophthalmic problem that affects diabetic people is called diabetic retinopathy (DR). Diabetic retinopathy (DR) can be categorized into two stages: proliferative diabetic retinopathy (PDR) and non-proliferative diabetic retinopathy (NPDR). When DR first appears, patients may not exhibit any symptoms of visual problems. However, as the condition progresses, it can result in blindness or vision loss, therefore early detection of DR is crucial to preserving patients’ vision. In 90% of cases, blindness can be avoided with early identification of DR. It is predicted that by 2045, there will be around 693 million people worldwide who have diabetes, according to data from the International Diabetes Federation [1]. The first clinical lesions of DR are called microaneurysms (MAs), which appear as reddish, round swellings in the capillaries that are either isolated or next to thin blood vessels. When assessing the progress of DR, the quantity of MAs is crucial. On the other hand, manual screening of MAs takes a long time and is trustworthy.

**Fig. 1:**
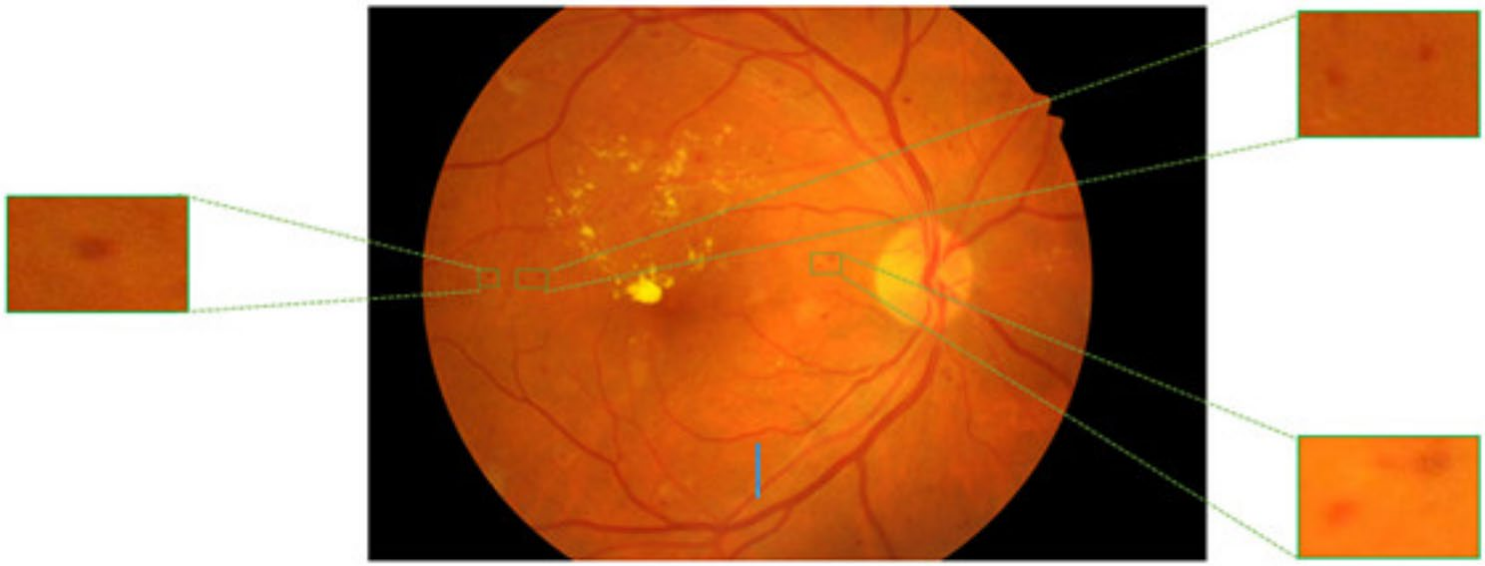
Microaneurysms in the fundus image. https://ieee-dataport.org/open-access/indian-diabetic-retinopathy-image-dataset-idrid

Therefore, computer-aided procedures are highly desired to save human labour and increase the accuracy of MA counting. A major challenge in medical image segmentation is the wide variance in lesions or illnesses’ size, shape, and location. In fundus images, MAs are visible as small, spherical, red dots that span a small number of pixels, with sizes varying from 15 to 60 μm. The four criteria listed below make diagnosing MA in fundus imaging difficult. Such as MA is a tiny objective, and the separation within the MA and non-MA areas is wildly out of proportion. The number of negative samples is significantly higher than the number of positive samples because the MA and non-MA regions are highly imbalanced due to the fundus image’s very tiny fraction of MA, as seen in Fig. 1. Furthermore, it is evident that MAs only take up a small portion of the fundus image’s pixels, which makes them challenging to recognize. Next to this, since some MAs are close to the blood vessels and resemble miniature blood vessels in both shape and color, they are especially vulnerable to interference zones. Furthermore, the classifier is confused by noise from behind and microscopic blood vessels because of the similarities with MAs. Finally, fundus images with poor contrast and hazy borders are full of MAs. Whereas other identical noisy zones are quickly and wrongly discovered, these MAs were simply ignored [2]. Even for seasoned ophthalmologists, manually identifying MAs can be difficult, time-consuming, and error-prone due to their small size, poor contrast, and amorphous appearance. The intricacy of differences in MA appearance prompts us to concentrate on creating a strong deep learning-based method for MA segmentation [3], which may find application in medical environments.

### Research contributions

The major contributions of this paper include.

- Proposed CBAM-AG-UNet, an enhanced U-Net framework for microaneurysm segmentation, which integrates the Convolutional Block Attention Module (CBAM) in the encoder and decoder to improve the feature extraction, and Attention Gates (AG) in skip connections to finetune the lesion-specific attributes, minimize background noise, and improve the fine details.
- CBAM in the encoder finds the most critical spatial and channel-wise features, enhancing initial feature representations, helpful for tiny lesion localization, such as microaneurysms (MAs).
- The three-fold attention decoding block is created with a multi-attention learning mechanism, incorporating AG, spatial attention for precise lesion localization, and channel attention for lesion-specific feature enhancement. This module captures fine-grained details and broader contextual information, significantly improving MA segmentation.
- The proposed framework works noticeably better than the current state-of-the-art techniques, as shown by broad investigations and assessments of benchmark datasets. The combination of AG and CBAM increases segmentation accuracy, feature refinement, and lesion localization, as verified by Dice and IoU metrics.

## Related works

Flaming et al. [4] and Quellec et al. [5] developed innovative image processing-based methods that can attain high accuracies relative to the MA detection challenge. These traditional techniques are known to be semiautomatic and to depend on well-crafted visual elements. Deep learning techniques can help mitigate this problem by systematically acquiring many filters or kernels using backpropagation-driven learning. For MA identification, Chudzik et al. [6] used patch-based fully convolutional neural networks (CNNs). Two pyramid feature extractors constitute a hierarchical “T”-shaped pyramid network that has recently been suggested in [7] for the detection of MAs. Using a shape suppression filter resolves the blood vessel interference that lowers the effectiveness of MA identification models. An efficient technique to identify MAs was presented by Zhang et al. [8] and was based on the idea of the local Fourier transform.

Other relevant research by authors [9–11], and [12] show how the authors have enhanced pre-existing models like DenseNet and YOLOv4 or used multistage techniques to identify MAs. Nevertheless, MA segmentation adds the ability to precisely identify early DR-affected regions on the retinal layer. In fundus imagery, there is a significant disparity in class between MA and non-MA regions, form variability, and visualization problems, accurately segmenting MAs is a challenging task. Tan et al. [13] examined the issue of highly unbalanced MA segmentation in the fundus images by using a ten-layer CNN. To provide impressive MA segmentation findings concerning specificity, sensitivity, and accuracy, the effective U-Net architecture [14, 15] for biomedical image segmentation has been altered in [16–19], and [20]. The incorporation of multiple residual learning as well as recurrent convolutional units in [16] and [18] substantially improved U-Net’s localization capabilities. To improve the quality of data sent via a skip connection, Xu et al. [19] added a feature fusion block to the encoder portion. To capture the finer characteristics of MA segmentation, an improved residual U-Net (ERUNet) that uses several up-sampling and down-sampling ways was proposed in [17]. Nevertheless, a considerable increase in the number of trainable model parameters results from these changes to the original U-Net.

It is also evident from the provided performance measurements that the effectiveness of MA segmentation needs to be further enhanced. To tackle this difficult task, we investigate the idea of visual attention mechanisms in this paper. Our studies have shown that selecting a low threshold [which relates to a high false positive rate (FPR)] for converting the final probability map to binary segmentation output can result in improved scores for standard metrics like accuracy, sensitivity, and specificity in [13–15], and [16]. Jingkun Chen et al. [21] presented a new semi-supervised technique that regularizes task-affinity consistency in the feature maps. An attention mechanism, which draws inspiration from the human visual system, focuses on the most discriminative areas of a deep network to increase its total efficacy [22]. The attention modules use a focused-on tasks dynamic selection mechanism to intelligently evaluate the convolutional features. The highly effective U-Net [14] or the subsequent residual U-Net [23] designs employ a skip connection to rapidly combine fundamental attributes from the contracting path with corresponding high-level features in the expansive path. Conversely, a skip connection with an attention component is referred to as an attentive-augmented connection, in which high-level features are concatenated with attended feature maps, and low-level features are adaptively enhanced as they move through the attention module [24]. Inspired by the success in natural language processing, a standard transformer known as Vision Transformer (ViT) with some alterations was used for CV in 2020 [25]. A small portion of the original image is used to linearly encode a series of cropping images that are fed into a ViT. Image patches are handled in an NLP framework in the same manner as tokens, or words. The transformer architecture has recently been used in the biomedical image processing field as well. The transformer architecture has recently been used in the biomedical image processing field as well. The ViT was used for anisotropic 3D medical image segmentation by Guo and Terzopoulos [26], with the self-attention model positioned at the base of the UNet architecture. Chen et al. [27] combined positional embeddings with picture patches to create a dual-channel transformer that could provide strong features. In this transformer approach, the two channels address tiny and big patches. A transformer-based model using a CNN and transformer for pathological image classification was proposed by Ding et al. [28]. In particular, local feature extraction is carried out by the CNN, and the global contextual information is extracted by the transformer. TransUNet [29] uses a Transformer as an encoder to investigate the global context information in medical images to segment them. Additionally, a pure Transformer with an encoder-decoder structure for multi-organ segmentation was proposed by Hu Cao et al. [30]. Transformers are incredibly effective at simulating long-range dependencies. But they can’t catch local features, which limits their performance. To combine the CNNs and Transformer into a new framework, certain recently pertinent techniques were used. For medical image segmentation, Li et al. [31] offer a dual encoding-decoding approach. The literature makes it clear that there are several problems with MA segmentation. One possible way to address the fundamental challenges in small and irregularly sized MA segmentation is to incorporate the idea of a visual system of attention as part of foundational U-Net design. Jingkun Chen et al. [32] proposed dynamic contrastive learning for medical image segmentation that leverages class confidence and confusion.

In contrast to previous works, we created a deeper CNN, AG (Attention gate), channel and spatial attention module-based novel triple-attention fundus image segmentation methodology that takes into account the most recent developments in the attention mechanism to address these challenges. In skip connections, the information gated from the encoded pathways is utilized to distinguish between unnecessary and noisy outcomes using AG attention. The spatial attention mechanism captures the spatial correlation between features, which enhances deep network performance.

## Materials and methods

**Fig. 2:**
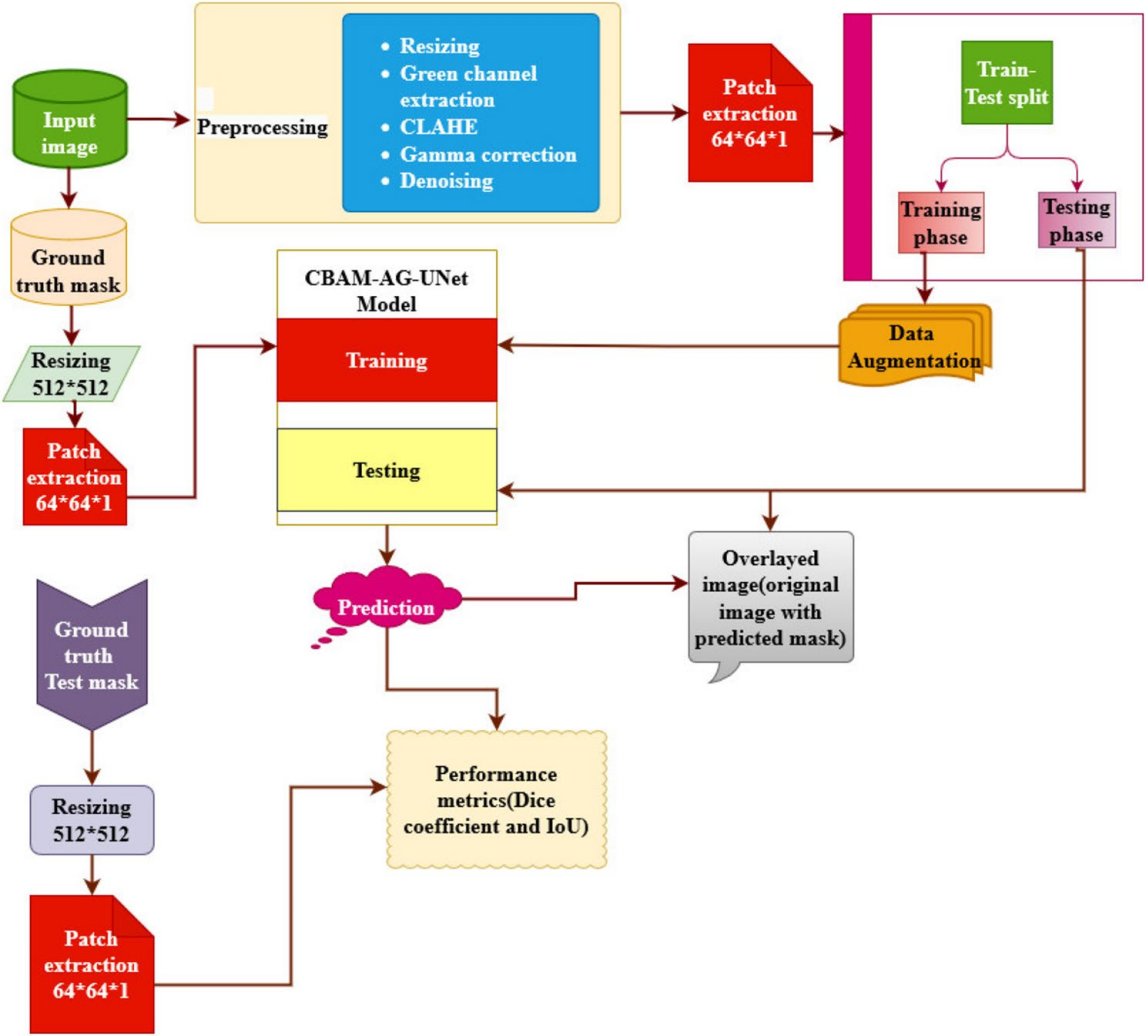
Proposed framework for MA segmentation

The proposed CBAM-AG-based U-Net framework of the MA segmentation includes input image acquisition, preprocessing, patch extraction, and augmentation as shown in Fig. 2. The function of each block is explained as follows.

### Dataset

The IEEE Data Port Repository is the public repository for obtaining the Indian Diabetic Retinopathy Image Dataset (IDRiD dataset), licensed under a Creative Common Attribution 4.0 license. This data descriptor 1 contains more detailed information regarding the data. We adhere to the Porwal et al. utilization of data permission. This dataset has 81 color fundus images, measuring 4288 × 2848 pixels. Of these, 64 have been employed for training and 17 for testing. Ophthalmologists have been marked 11,716, 1903, 150, and 3505 related locations in IDRiD as EX, HE, SE, and MA, respectively. In this research work, we concentrate especially on MAs because our work is purely related to MA segmentation. The total number of images is 81, 64 for training, and 17 for testing. As mentioned earlier there are 3505 locations related to MA. To conduct experiments and replicate the comparison approaches on IDRiD, we use training sets to train the models and testing sets for testing. To assess our CBAM-AG-UNet, we use this dataset and only focus on consideration of the pixel-level visual annotations (i.e., 81).

### Data pre-processing

Pre-processing is done on the original fundus images to improve the quality of the images for training. The RGB fundus image’s green channel is used for additional processing because it has the best contrast. When the contrast of the acquired image is excessively low, it is challenging to distinguish and isolate the objects of importance. Image improvement is thus a crucial stage that comes before learning.

The initially collected images (2848 × 4288 × 3) in the IDRiD dataset were cropped out (2848 × 3450 × 3) applying the batch processing approach since they contained a black backdrop and unnecessary information. Then, to increase the deep learning model’s input size, it is resized to a fixed size of 512 × 512 × 3. To offer equally sized images for automated analysis, the dataset’s images are scaled. This will make the recommended approach more efficient and reduce its processing time. This automated approach will not work without the help of image scaling. Next, we will look at the original fundus image, which has three channels and is represented as f (p, q). We will now investigate the original fundus image, denoted as f (p, q), which is made up of 3 channels: red $\:{f}_{r\:}\left(p,q\right)$, green $\:{f}_{g\:}$p, q), and blue $\:{f}_{b\:}\left(p,q\right)$ is represented in Eq. (1).

1 $$f\left({p,q} \right) = [\kern-0.15em\left[[ {\left. {{f_{r\,\,}}\left({p,q} \right),{f_{g\,\,}}\left({p,q} \right),{f_{b\,\,}}\left({p,q} \right)} \right]} \right.\,$$

$\:\left\{{f}_{g\:}\right(p,q)$ has better contrast}

It is evident from examining the fundus image of all of the channels that the green channel has a higher contrast between the backdrop and the microaneurysms. To enhance contrast, the entire image is separated into grids that do not overlap the dimensions (8, 8) in CLAHE (contrast-limited Adaptive Histogram Equalization). Then, each tile is exposed to histogram equalization. The greatest amount of contrast improvement that can be performed on each grid of the processed image is determined by the clip limit of CLAHE. The technique known as CLAHE [33] is employed to achieve a consistent decrease in noise amplification and equalization of intensity. Gamma correction (GAMMA) is performed using Eqs. (2) and (3), respectively, to modify the augmented image’s overall brightness and lessen the overexposure conditions.

2 $$\:{f}_{g\_clahe\_gamma}=GAMMA\left({f}_{g\_clahe}\:,\:\gamma\:=0.9\right)$$

where

3 $$\:GAMMA\left(R,\gamma\:\right)=\left\{{\left(\frac{R}{{R}_{max}}\right)}^{r}\right\}*{R}_{max}$$

This research displays better denoising performance for medical images and effectively eliminates noise using a fast Non-Localized Means filter. Next to this patch extraction. In many image-processing applications, patch extraction is a crucial preprocessing step. It offers a collection of locally smaller images, or patches, that are chosen at random to represent an image. We first pre-process the fundus image before proceeding with patch extraction. Table 1 shows the number of images used for training and testing. This study substitutes patch-based training for global image-based training to address data scarcity. Regarding the need for pixel-level annotations in medical image segmentation, data scientists are the most concerned. For actual deep neural network training, it is therefore always difficult to gather a substantial amount of labeled data, and most techniques that employ deeper models tend to perform below expectations. To a certain extent, certain techniques—like augmentation and transfer learning—can work with less data. This method extracts numerous regions from one image to deliver multiple instances with more data. Microaneurysms are small and sparsely distributed lesions that can appear anywhere in the fundus image, making region-based detection unreliable. Unlike larger lesions that may be localized, microaneurysms do not have a fixed position, and an object detection approach may fail to capture all instances, leading to missed detections. To achieve full segmentation, we implemented a patch-based method that divided the image into tiny regions to ensure that each area could be analyzed identically. This method ensures that no lesions are ignored while maintaining spatial properties. In addition, our three-fold attention strategy enhances feature extraction to enhance segmentation rather than adding a detection stage. Patch-extracted training images have undergone data augmentation to increase the diversity of data. Data augmentations used are random rotation 90^0,^ Horizontal and vertical flip.

**Table 1:** Image distribution for MA segmentation for training and testing

Original images	Training images (512 × 512)	Testing images (512 × 512)	No. of training samples (64 × 64 patch extracted)	No. of testing samples (64 × 64 patch extracted)	No. of training samples after augmentation
81	64	17	4096	1088	16,384

### Proposed CBAM-AG-based UNet architecture

**Fig. 3:**
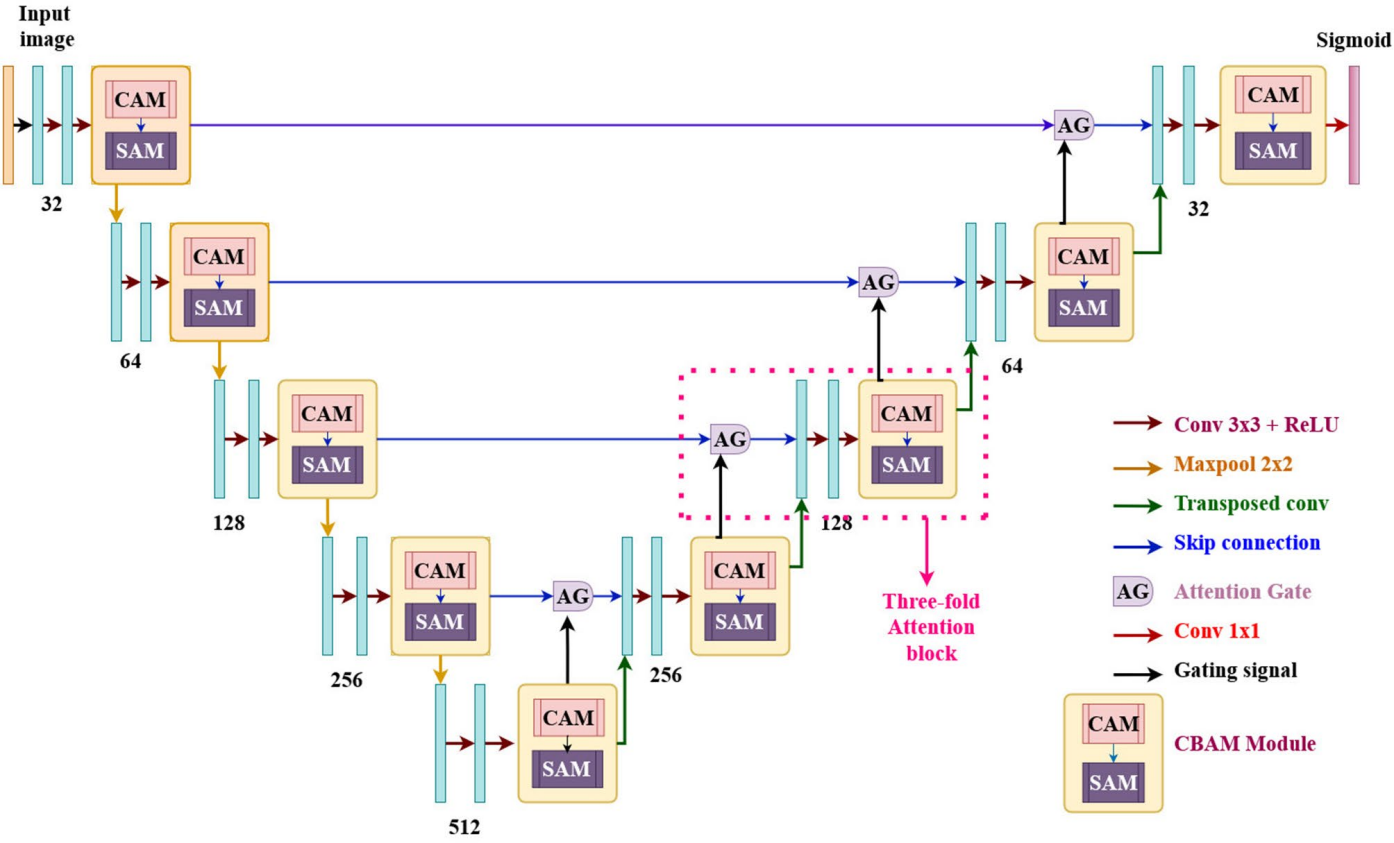
The architecture of the proposed CBAM-AG-UNet model

Building on the Convolutional Block Attention Module (CBAM) [34], Attention U-Net [24], and U-Net [14], we suggest CBAM-AG-UNet shown in Fig. 3 to improve microaneurysm (MA) segmentation in fundus pictures. We explain in detail about each part of the network below.

The CBAM-AG-UNet encoder-decoder structure is U-shaped. To improve feature learning, a CBAM module is added after a structured convolutional block in each encoder step. To improve segmentation, the decoder includes a three-fold attention decoding block that records contextual information as well as fine-grained data. Convolutional layers and Rectified Linear Unit (ReLU) activations constitute each convolutional block. To improve feature extraction, the network doubles the amount of feature channels in the encoder path at each downsampling stage. Using 2 × 2 transposed convolutions, up-sampling is carried out in the decoder route to gradually recreate the segmentation map. Encoder and decoder feature maps are connected by skip connections, allowing for feature reuse. Lesion segmentation is improved by these connections as they go through the three-fold attention decoder block. A 1 × 1 convolution and a sigmoid activation function are used to create the final segmentation map, which yields the microaneurysm segmentation output.

#### CBAM module

**Fig. 4:**
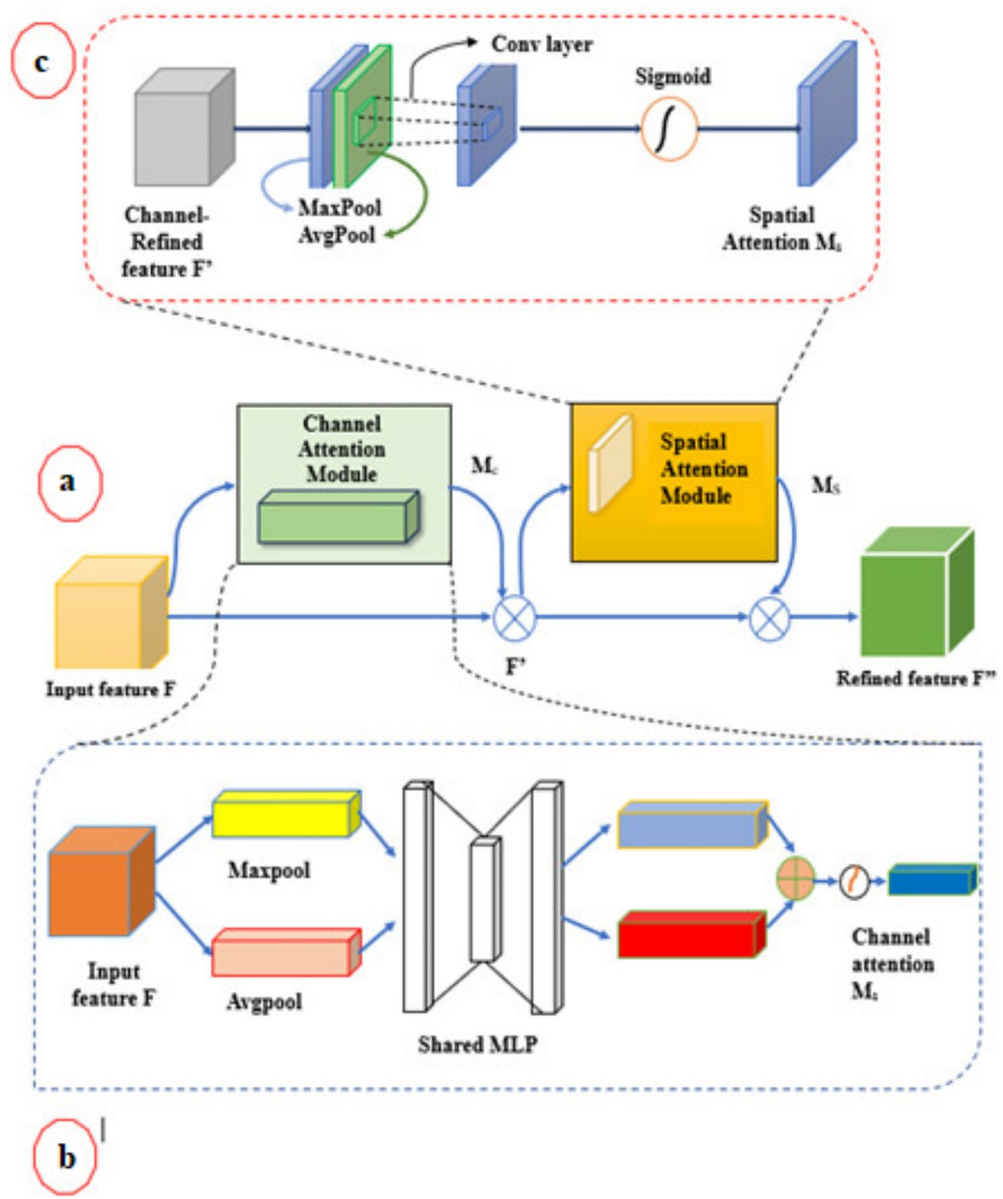
(**a**). Convolutional block attention module (**b**). Channel attention module (**c**). Spatial attention module

To obtain more precise information about visual features, we employ CBAM [29] as the network’s attention module. Attention modules have been employed in recent research to help CNNs concentrate on more significant elements from input images and avoid getting lost in less significant ones. To enhance the weights of informative elements in the channel and beneficial attributes in the space, CBAM combines channel attention and spatial attention, as shown in Fig. 4. The resulting combination indicates that key properties in both channels and spatial locations are given priority by the network. The channel attention generates a 1D attention map $\:{M}_{c}\in\:{R}^{C\times\:1\times\:1}\:,$ which prioritizes the most important feature channels. Spatial attention produces a 2D attention map $\:{M}_{s}\in\:{R}^{1\times\:H\times\:W}$, which enhances the focus on key spatial regions. The attention mechanisms are mathematically expressed as

4 $$\:\text{C}\text{h}\text{a}\text{n}\text{n}\text{e}\text{l}\:{F}^{{\prime\:}\:}={M}_{c}\left(F\right)\otimes\:F$$

and

5 $$\:Spatial\:{F}^{{\prime\:}{\prime\:}}={M}_{s}\left({F}^{{\prime\:}}\right)\otimes\:{F}^{{\prime\:}}$$

where ⊗ denotes element-wise multiplication.

The channel attention values are spread across the spatial dimension, enhancing the importance of global features. Next, the spatial attention values are applied across feature channels, ensuring a more precise spatial focus. The final, enhanced feature representation is denoted as F′′. Each attention map’s computational process is depicted in Fig. 4, with detailed information about the individual attention modules provided in the following sections.

##### Channel attention module

The Channel Attention Module uses the relationships between different feature channels to generate a channel attention map, enabling the network to concentrate on key elements in the input image. By treating each channel in a feature map as a feature detector, channel attention ensures that the most crucial channels are given priority. To infer finer channel-wise attention, max-pooling collects yet another significant hint regarding distinguishing object attributes. As a result, we simultaneously apply average-pooled and max-pooled characteristics. Instead of using each feature separately, we experimentally showed that utilizing both significantly increases the representation ability of networks. To generate two different spatial context descriptors, $\:{F}_{avg\:}^{c}$and $\:{F}_{max}^{c}$, which represent average-pooled features and max-pooled features, respectively, we initially combine the spatial information of a feature map by applying both average-pooling and max-pooling procedures. After that, both descriptors are sent to a common network, which creates our channel attention map $\:{M}_{c}\in\:{R}^{C\times\:1\times\:1}$. A multi-layer perceptron (MLP) with a single hidden layer constitutes the shared network. The hidden activation size is set to $\:{R}^{c/r\times\:1\times\:1},$ where r is the reduction ratio, to minimize parameter overhead expenses. Following the application of the shared network to every descriptor, we use element-wise summing to combine the resultant feature vectors. To summarize, the channel attention is calculated as follows:

6 $$\eqalign{{M_{c }}\left(F \right) = & \sigma \left({MLP\left({AvgPool\left(F \right)} \right) + MLP\left({MaxPool\left(F \right)} \right)} \right) \cr = & \sigma \left({{\omega _{1 }}\left({{\omega _{0 }}\left({F_{avg }^c} \right)} \right) + {\omega _{1 }}\left({{\omega _0}\left({F_{max}^c} \right)} \right)} \right) \cr} $$

Where $\:{\omega\:}_{0}\in\:{R}^{c/r\times\:c}$, $\:{\omega\:}_{1}\in\:{R}^{c\times\:c/r}$, and σ represents the sigmoid function. The MLP weights, $\:{\omega\:}_{0}$ and $\:{\omega\:}_{1}$, are shared for both inputs, and ReLU activation is applied after $\:{\omega\:}_{0}$.

##### Spatial attention module

The Spatial Attention Module makes use of the inter-spatial connections of features to highlight the “where” the key areas of an image are identified by spatial attention, as opposed to channel attention. Average pooling and max pooling operations along the channel axis are combined to produce two different contextual descriptors, which are then used to calculate spatial attention. By aggregating the channel-wise information, we produce two 2D feature maps, $F_{avg}^{s} \in {R^{1 \times H \times W}}$ and $\:{F}_{max\:\:}^{s}\in\:{R}^{1\times\:H\times\:W}$, representing average-pooled and max-pooled features, respectively. These are then concatenated and passed through a 7 × 7 convolutional layer to generate the spatial attention map $\:{\:M}_{s}\left(F\right)\in\:{R}^{1\times\:H\times\:W}$ which determines where to focus in the input feature map. The spatial attention is calculated as follows:

7 $$\eqalign{{M_s}\left(F \right) = & \sigma ({f^{7 \times 7}}\left({\left[ {AvgPool\left(F \right);MaxPool\left(F \right)} \right)} \right) \cr = & \sigma \left({{f^{7 \times 7}}\left({\left[ {F_{avg }^c; F_{max }^s} \right]} \right)} \right) \cr} $$

Where $\:{f}^{7\times\:7}$ is a convolution operation with a 7 × 7 filter size, and represents the sigmoid function.

##### Sequential vs. parallel arrangement

Two attention modules—channel and spatial—calculate complimentary attention to an input image by concentrating on “what” and “where,” respectively. The two modules are capable of being arranged either sequentially or in parallel in light of this. We discovered that a sequential arrangement outperforms a parallel arrangement. Outcome of our experiment indicates that the channel-first arrangement is marginally superior to the spatial-first for the sequential process layout, thereby significantly enhancing feature extraction and segmentation performance.

#### Three-fold attention module

**Fig. 5:**
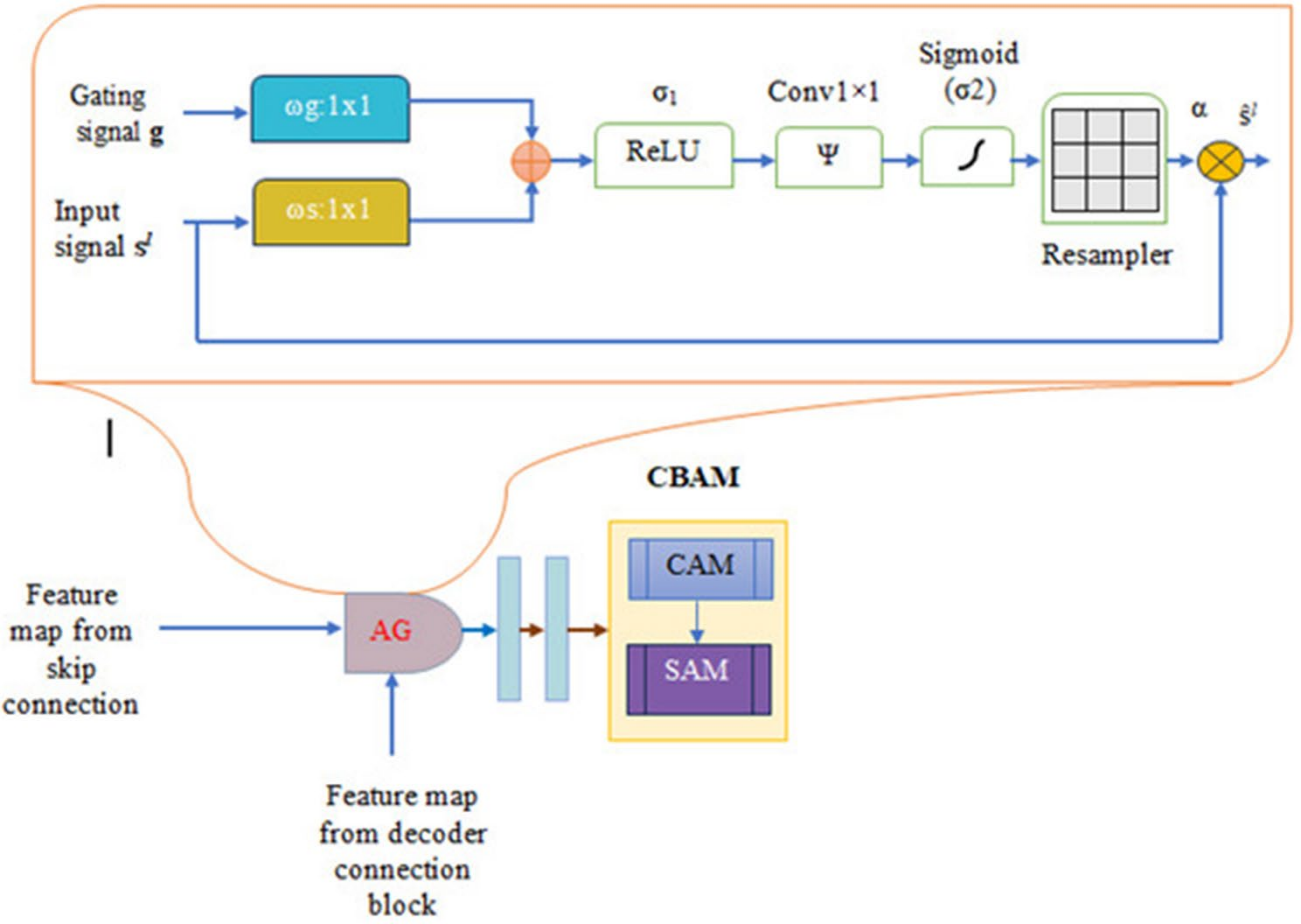
Three-folded architecture

The three-fold attention decoder module shown in Fig. 5 improves the up-sampling (decoding) process while also offering specific and spatial data. By using skip connections, this section incorporates features derived from the encoder path, adding more information to the feature map. Both spatial and channel attention are components of the dual attention mechanism that makes up the decoder block. Following Attention Gate (AG) processing on the concatenated feature map, the combined output is normalized using a typical 3 × 3 convolution operation. By concentrating on channel interactions to enhance feature representations and identifying spatial correlations between features to draw attention to the pertinent areas, this three-pronged strategy dramatically boosts performance, as shown by Hu et al., then concentrating on the most crucial regions by making decisions based on contextual knowledge.

##### Attention gate

Attention Gates (AG) [24] are employed in U-Net designs [14] to highlight the most significant details in an image. They do this by using incremental attention to compute attention coefficients (α) which scale the given input features significantly. Therefore, throughout training, vital regions are given additional weight and attention. The gating signal (g), which comes from the previous layer of the upsampling/decoder section and provides coarse information, and the encoder features, which are passed in via skip connections from the downsampling/encoder section and provide more detailed features, are the two inputs obtained by the attention gate in the U-Net architecture design. During training, this configuration guarantees that the model gives priority to the most important regions of the input image. By giving input characteristics attention coefficients (α), the AG mechanism helps the model focus on significant areas while reducing the impact of noisy or unnecessary parts. By integrating these modules with Attention U-Net’s CBAM block, the network may concentrate on high-level features, increasing segmentation accuracy.

By eliminating unnecessary information and emphasizing the most important areas for the task, the AG automatically learns to recognize desired structures of different shapes and sizes in medical imagery. In the forward progression, the model adjusts activations in the skip connections to identify important regions; in the backward motion, gradients from the background areas are down-weighted to make sure the model updates parameters based on task-relevant spatial regions. By using trilinear interpolation to compute the attention coefficients (α) for the reproduced grids and then scaling the input features (sl), the Attention Gate mechanism improves the representation capability of the U-Net with little computational overhead. The gating signal (g) filters attributes at more fine scales and chooses the most important areas. Our model effectively retains crucial features after 32× and 16× downsampling by employing skip connections, CBAM, and attention gates. Skip connections restore spatial details by directly transferring high-resolution features to subsequent layers. CBAM emphasizes key lesion features through channel and spatial attention, ensuring that important information is preserved even at lower resolutions. Attention gates, which target tiny structures like microaneurysms, improve feature recovery even further. Additionally, to improve feature representation and guarantee accurate segmentation even in the face of downsampling, the three-fold attention block integrates several attention techniques. These combined techniques enhance tiny lesion segmentation and avoid feature loss.

In the end, the triple attention mechanism combined with the attention gates (AGs) to improve the segmentation by concentrating on key features from the channel and spatial point of view, using the coarse scale information from the gating signal to remove the insignificant details combining the attributes and ensuring both forward and backward passes highlight important regions by adjusting neuron activations and gradients. This method helps the U-Net architecture learn more effectively, increasing segmentation accuracy and robustness.

#### Loss function

The cross-entropy (CE) loss function is often used in image segmentation research since it measures the information difference between the prediction and ground truth areas. An important indicator of the cross-entropy relationship between the ground truth distribution p and the probability distribution q is the average number of bits of the coding length required for the ground truth distributed p to identify a sample. The average cross-entropy of all pixels is typically used to compute the cross-entropy loss in image segmentation tasks. $\:{q}_{i}$, $\:{p}_{i}$ denotes the predicted segmentation and ground truth of voxel I, respectively. K is the number of voxels in the image I.

8 $$\:{L}_{BCE}=\:-\frac{1}{K\:}\sum\:_{i=1}^{K}\left[{p}_{i}log{q}_{i}+\left(1-{p}_{i}\right)\text{log}\left(1-{q}_{i}\right)\right]$$

## Performance metrics

The metrics used to evaluate the reliability of the suggested method are illustrated in this section; these metrics are commonly used to evaluate the effectiveness of semantic segmentation. We demonstrate how precisely the segmentation generated by the algorithm matches the ground truth and give their numerical techniques for determining its success rate. True positive (TP), True negative (TN), False positive (FP), and False negative (FN) are examples of metrics that are computed at the pixel level.

### Accuracy

It is referred to the ratio of correct predictions to the all-predicted pixels.

9 $${\rm{Accuracy}} = {{{\rm{TP}} + {\rm{TN}}} \over {{\rm{TP}} + {\rm{FP}} + {\rm{FN}} + {\rm{TN }}}}$$

### Area under the curve - receiver operating characteristic (AUC-ROC)

For segmentation, the pixel-wise predictions can be treated as binary classification results. It is especially beneficial when working with imbalanced datasets. To measure the AUC-ROC; we plot the True Positive Rate (TPR) against the False Positive Rate (FPR) with different threshold values and compute the area under the curve.

10 $$\:True\:positive\:rate=\frac{TP}{TP+FN}$$

11 $$\:False\:positive\:rate\:=\frac{FP}{FP+TN}$$

### Dice similarity coefficient

The dice similarity coefficient is a frequently employed statistic in image segmentation that assesses the degree of similarity between a ground truth mask and a predicted segmentation mask. We measure the overlap of the two masks.

12 $$\:\text{D}\text{i}\text{c}\text{e}=2\times\:\frac{\text{P}\times\:\text{S}\text{E}}{\text{P}+\text{S}\text{E}}$$

### Intersection of union

It is a metric used to assess how well the segmentation results(S) and the ground truth mask(G) overlap.

13 $$\:IoU=\frac{|G\cap\:S|}{|G\cup\:S|}$$

## Experimental results

### Simulation environment

For deep learning applications like MA segmentation in medical images, a simulation environment with a T4GPU, Keras version 2.15.0, and Python 3 as the deep learning framework works quite well. To train the network, we set the hyperparameters and employed the Adam optimizer with the initial learning rate set to 1 × 10 − 3 and adjusted using the ReduceLROnPlateau method. The validation loss is tracked, and after five epochs without progress, the learning rate shrinks in half, but it never falls below the minimum value of 1e-5. We train and evaluate the IDRiD dataset on an initialized network. While training, we make use of a patch size of 64 and provide standard augmentation methods such as random rotation and flip. There are 40 training epochs followed by a batch size of 16 (See Table 2).

**Table 2:** Hyper-parameter values

Parameter	Values
Batch size	16
Image resolution	512 × 512
Optimizer	Adam
Optimal learning rate	0.001 to 0.00001
Patch size	64 × 64
Epochs	40

### Training and testing of the model

**Fig. 6:**
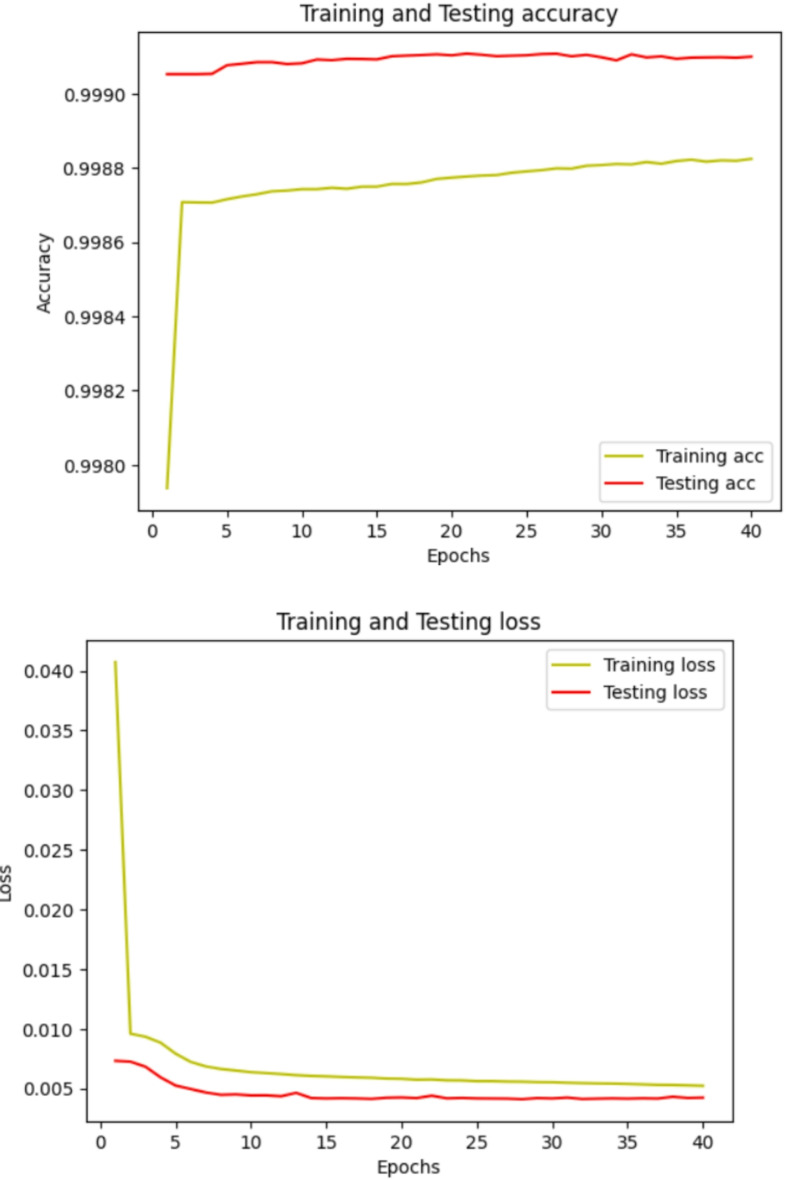
Training and testing curves for model accuracy and loss

Model accuracy with loss validation techniques is employed to evaluate the model’s training procedures. Over the whole training dataset, one epoch is one full iteration. The learning method processes every training example once throughout an epoch. The dataset is frequently split up into smaller batches, and the model iteratively adjusts its parameters for each batch for each epoch. When the model’s accuracy reduces and its loss improves throughout the training phase, it is overfitting rather than learning. When accuracy enhances and loss shrinks, the model is learning. Figure 6 illustrates the accuracy and loss of the suggested model for the test and train information. The suggested model was given 40 epochs to finish and used an entire set of 16,384 image patches for training and 1088 images for testing. Test outcomes during segmentation show that the model executed very effectively in both training and testing. The training and testing accuracy of the proposed model achieved 99.88 and 99.91 for 40 epochs respectively.

### Pre-processing output for MA segmentation

**Fig. 7:**
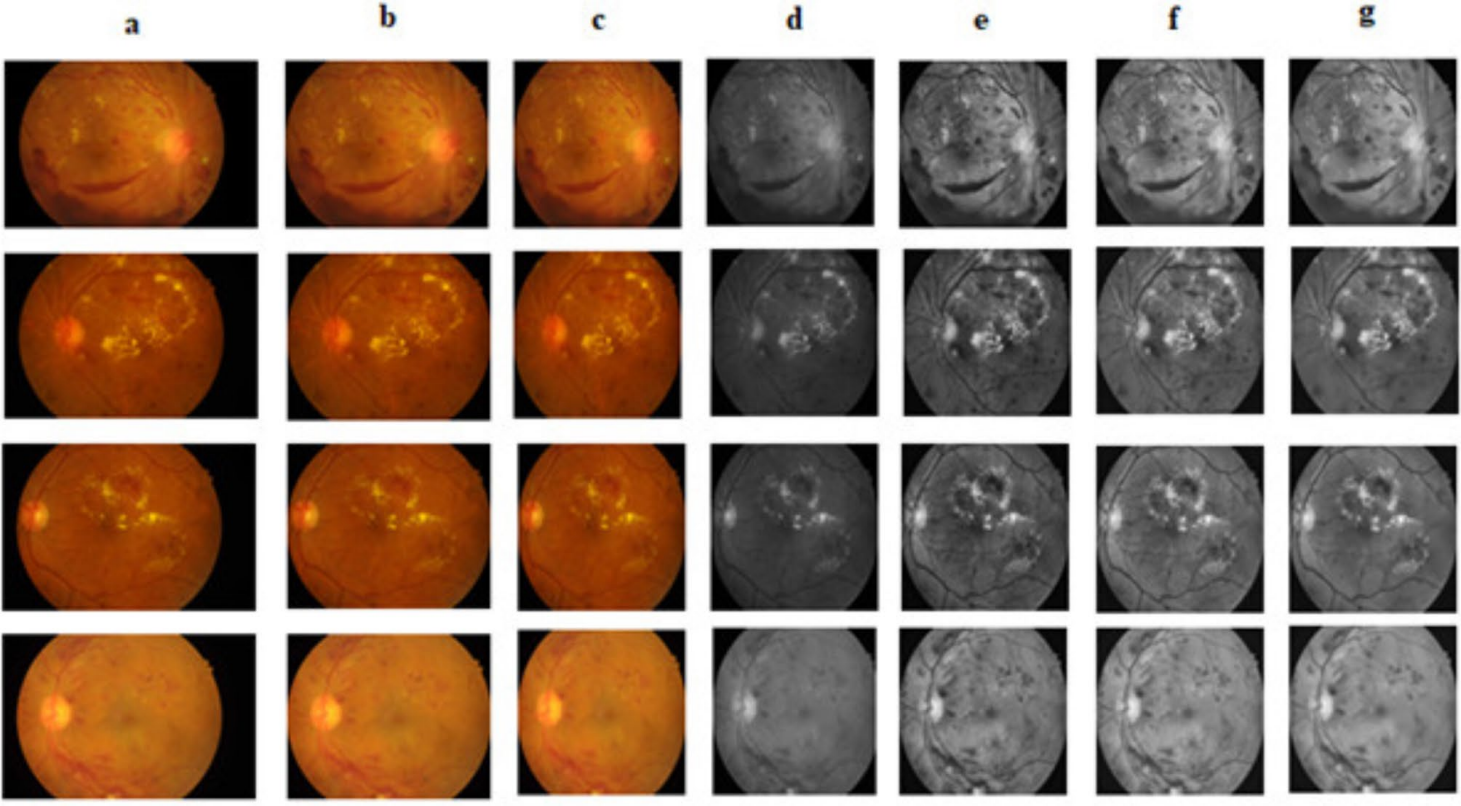
(**a**). Input image (**b**). Cropped image (**c**). Resized image (**d**). Green channel (**e**). Gamma corrected image (**f**). De-noised image

Pre-processing is a multi-step procedure that gets raw data ready for a deep learning model. By eliminating extraneous portions from a rectangular zone that is relevant within an image, cropping increases efficacy by highlighting key elements. Image specifications are standardized through resizing (512 × 512), which makes processing more consistent. Enhancing key attributes in the green spectrum, that is, extracting the green channel which is particularly helpful in medical imaging improves model performance in tasks like segmentation. Local contrast is improved using CLAHE (Contrast Limited Adaptive Histogram Equalization), which makes features simpler to see and detect by the model. To improve feature identification, gamma adjustment brings the brightness of an image closer to its normal level. Finally, by lowering noise in the image, denoising enhances quality and results in more precise feature extraction. These procedures work together to convert unprocessed images into easier-to-understand and useful information for deep learning. The results of various pre-processing methods are depicted in Fig. 7.

**Fig. 8:**
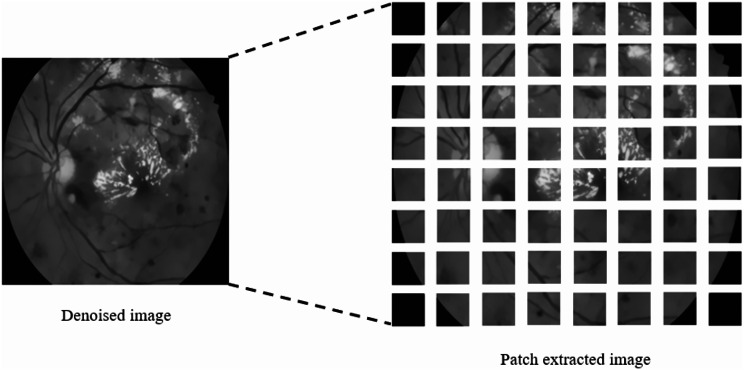
Sample denoised image and patch extracted from the denoised image

**Fig. 9:**
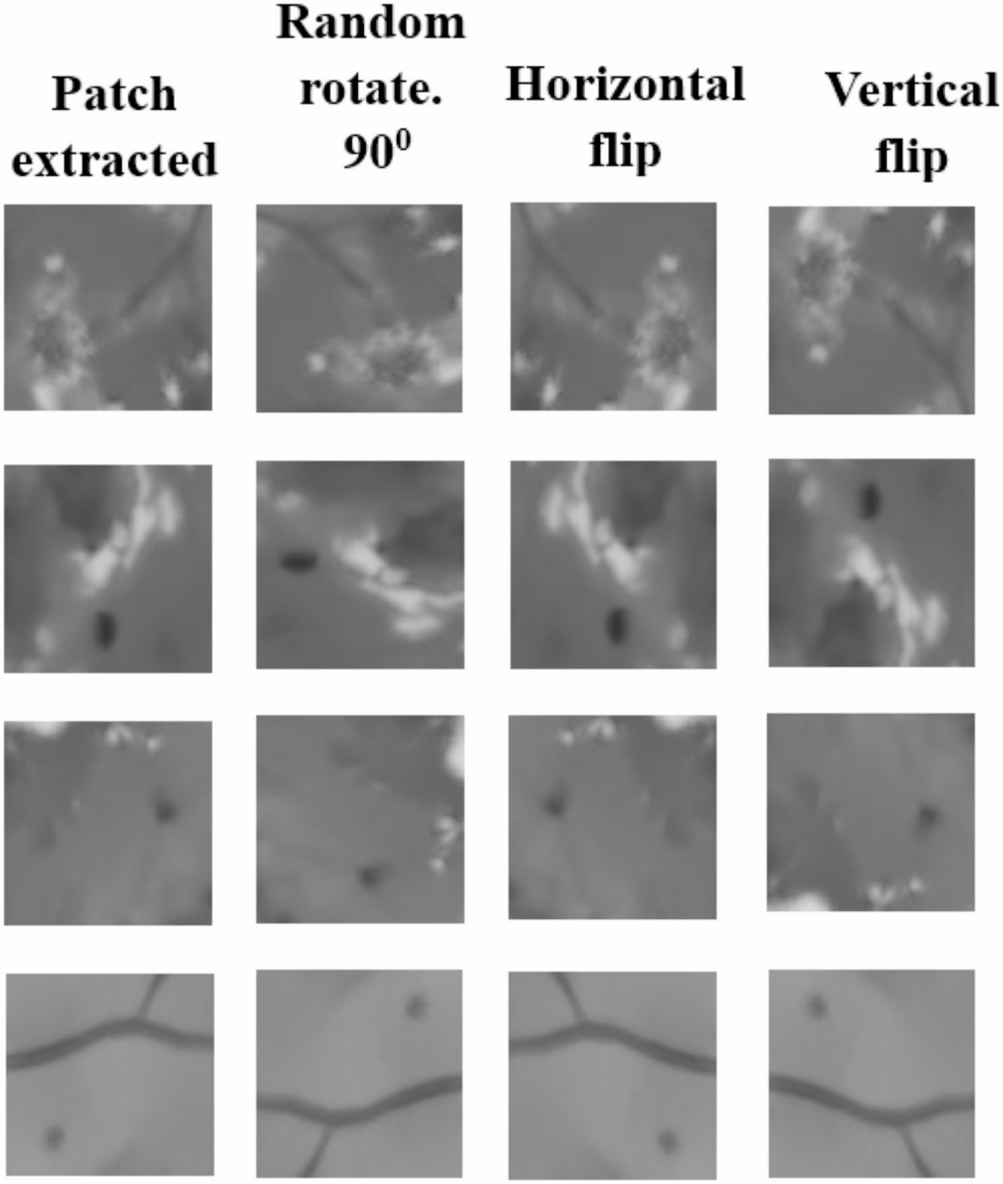
Data-augmented patches. From left to right (**a**) patch extracted (**b**) random rotate 90^0^ (**c**) horizontal flip (**d**) vertical flip

Next to denoising, patch extraction helps deep learning models to develop and operate more precisely by breaking up the original images into smaller, more useful portions. It’s an essential preprocessing step that raises the entire ability and efficacy of the information in the image. The denoised image has a dimension of 515 × 512, which can extract the 64 × 64 patches, creating a primary grid of 64 patches. Instead of using the whole image, a patch gives better results for tiny objects like segmenting tasks like microaneurysms. By concentrating on small patches, the model is better able to understand the background and foreground information. The sample denoised image and the corresponding patch-extracted image for this denoised image is shown in Fig. 8. Then after extracting the patches, it is very low in number (4096) to train the model, hence we have to increase the diversity of the training dataset we applied data augmentation like random rotate 90^0^, horizontal and vertical flip correspondingly. Finally, we obtained 16,384 images for training the proposed model. Figure 9 represents the data-augmented patches.

### MA segmentation results

**Fig. 10:**
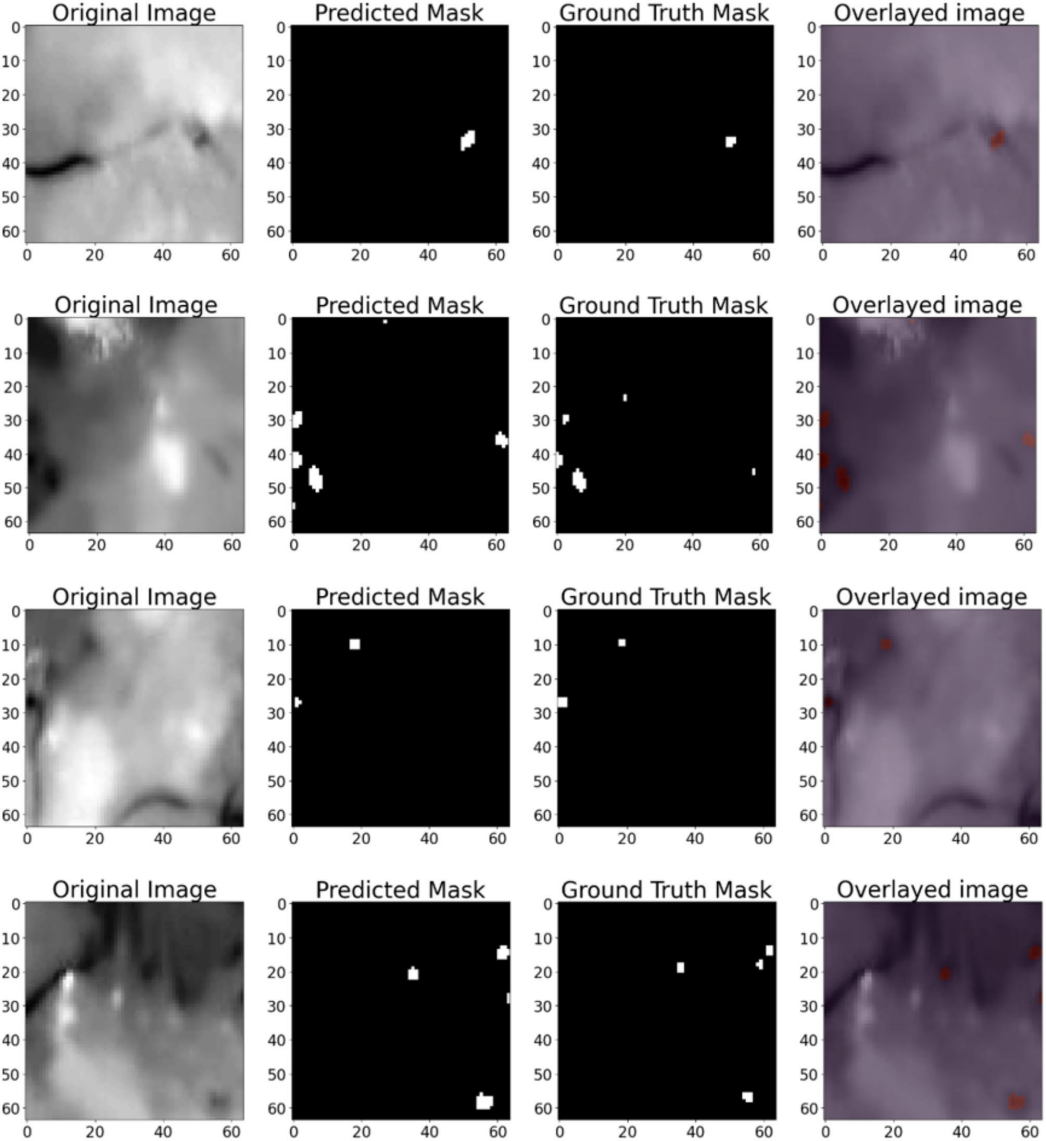
Segmentation results of IDRiD dataset for microaneurysms showing (**a**). Original image, (**b**). Ground truth mask, (**c**). Predicted mask and (**d**). Overlayed image

We examined the suggested approach using the IDRiD fundus image dataset. We contrasted it with state-of-the-art MA segmentation and traditional image processing techniques to show how effective the CBAM-AG-UNet is at segmenting small MAs. The appearance results of segmentation from our suggested segmentation network on a few fundus image patches from the IDRiD dataset are shown in Fig. 10. The current deep-learning techniques yield poor MA segmentation results because they are unable to maintain precise boundaries. We tested the CBAM-AG-UNet model’s performance on the IDRiD dataset for segmenting microaneurysms (MAs) in fundus images, comparing it to top-of-the segmentation approaches and standard image processing methods. Our model obtained a remarkable 99.91% accuracy, indicating its ability to classify a large number of pixels accurately. The Dice Score of 0.865 and IoU of 0.758 indicate it reliably recognizes and segments MA regions, highlighting the difficulty of distinguishing tiny and scattered MAs. However, the model’s high AUC-ROC score of 0.996 demonstrates its ability to discriminate MAs from their surroundings across various levels. These findings emphasize the CBAM-AG-UNet’s ability to perform precise MA segmentation, which is critical for early diabetic retinopathy identification, while also indicating areas for additional improvement in obtaining finer segmentation details. In contrast, when compared to the ground truths, the suggested approach in Fig. 10(c) produces reliable results with high precision in patches with one or several MAs. We then present the quantitative findings to assess our CBAM-AG-UNet’s performance more precisely.

## Ablation study

An ablation study assesses the effects of structural modifications and hyperparameter settings on segmentation performance. The structural ablation focuses on variations in network design, while hyperparameter tuning explores aspects like batch size, and patch size. This study aims to determine the optimal configuration for achieving superior segmentation results.

### Structural ablation

The performance of CBAM-AG-UNet was evaluated with and without Attention Gates (AG) and for different bottleneck depths. The proposed CBAM-AG-UNet, with a 512-filter bottleneck, gives the utmost performance, with a Dice score of 0.865, an IoU of 0.758, and an inference time of 0.3392 s, which is less compared to the other scenarios. We clearly stated that the attention gates allow the model to concentrate on the most important regions of the input, minimizing the amount of calculation required and improving the processing time, and memory usage was 17.11 MB. When AG was removed from the skip connections, the model’s performance decreased extremely to a Dice score of 0.715, an IoU of 0.652, an inference time of 1.5184 s, and memory usage of 18.41 MB, which underlines the critical role of AG in improving segmentation accuracy. Moreover, reducing the bottleneck depth to 256 filters further reduced performance (Dice score: 0.698, IoU: 0.645, inference time of 0.3556 s, and memory usage was 17.35 MB). when AG is removed from skip connections with 256 as a bottleneck, further diminishing the performance (Dice score: 0.612, IoU: 0.598, inference time of 1.7214 s, and memory usage was 18.72 MB). From this, we observed the necessity of a deeper feature extraction process for effective lesion segmentation. These findings are described in Table 3, then validate that the inclusion of AG in skip connections and a deeper bottleneck are essential for achieving superior segmentation.

**Table 3:** Structural ablation analysis of the proposed model

Ablation	Dice	IoU	Total inference time(sec)	Memory usage during inference (MB)
Unet + CBAM + AG with 512 as a bottleneck (Proposed)	0.865	0.758	0.3392	17.11
Unet + CBAM with 512 as a bottleneck	0.715	0.652	1.5184	18.41
Unet + CBAM + AG with 256 as a bottleneck	0.698	0.645	0.3556	17.35
Unet + CBAM with 256 as a bottleneck	0.612	0.598	1.7214	18.72

### Hyperparameter experiments with CBAM-AG-UNet

For hyperparameter tuning various batch sizes and patch sizes were tested to examine their performance on segmentation quality. A batch size of 16 showed the highest achievement (Dice: 0.865, IoU: 0.758), whereas increasing it to 32 led to a performance drop (Dice: 0.685, IoU: 0.630), likely due to unstable weight updates. Furthermore, 64 × 64 patches yield better results (Dice: 0.865, IoU: 0.758) than 128 × 128 patches (Dice: 0.678, IoU: 0.625), indicating that smaller patches preserve minute lesion features. As indicated in Table 4, these results emphasize how important it is to choose hyperparameters properly to attain segmentation accuracy. Then the Dice loss is applied to the proposed method which decreases the performance (Dice: 0.412, IoU-0.402) and for Focal loss also performance is degraded such as (Dice:0.346, IoU: 0.312). Finally, the learning rate scheduler, the model automatically adapts the optimal learning rate from 0.001 to 0.00001enhance the convergence and prevents overfitting. These findings denote the importance of carefully selecting hyperparameters to achieve a balance between training efficiency and segmentation accuracy, as described in Table 4.

**Table 4:** Hyperparameter ablation study of the proposed model

Hyperparameter variation	Dice	IoU
Batch size 16 (Proposed)	0.865	0.758
Batch size 32	0.685	0.630
Patch size 64 × 64 (Proposed)	0.865	0.758
Patch size 128 × 128	0.678	0.625
Focal loss	0.346	0.312
Dice loss	0.415	0.402

This ablation investigation demonstrates that segmentation performance is significantly improved by structural enhancements such as the addition of AG and a deeper bottleneck. The model’s efficiency is further enhanced by the meticulous selection of hyperparameters, such as batch size, and patch size.

## Discussion

The ultimate goal of this study is to demonstrate that the suggested model outperforms the most effective deep learning algorithms for segmenting microaneurysms. The CBAM-AG-UNet model’s accuracy in detecting microaneurysms has improved. The majority of present U-shaped approaches are good at segmenting huge objects, but they are not good at detecting minute items, which are very common in eye disorders. The feature relationships in the hidden layers are weaker than their initial inputs because of the downsampling and upsampling procedures in U-Net. This leads to a loss of visual information and lower segmentation efficiency for the minute lesions, comparable with segmentation models. The proposed model outperforms existing methods in segmentation, notably targeting background and foreground pixels, as well as the Dice coefficient as 0.865 IoU as 0.758, and AUC as 0.996. The segmentation imagery shows that the suggested model’s segmentation findings are more accurate with deep and exact feature extraction.

The suggested model distinguishes itself from the previous frameworks, MCA-UNet [35], Galance Seg [36], MSAG [37], TC-UNet [38], AFTE [39], PMC Net [40], L-Seg [41]. MCA-UNet combined semantic information at various levels using the multi-scale feature fusion technique and gathered pertinent global dependencies using the cross-co-attention module. Incorporating gaze maps acquired while doctors view images, Galance Seg allows for top-down attention-driven coarse lesion localization. Using indication points produced from saliency maps, bottom-up attention guiding further optimizes this process. Furthermore, a DKF module is used to improve the segmentation of unclear MAs that the basic model generates. Significantly, MSAG is excellent at identifying a wide variety of lesion sizes and exhibits a balanced ability to identify across lesion types, both of which are essential for thorough DR assessment. In TC-Net a new feature fusion technique called LLCS (long-range dependency concatenation strategy), combines the various feature maps from the Transformer and CNN branches to produce better segmentation results. To include both vascular and lesion details in the primary generator, AFB(attention fusion block) integrates self-attention and cross-attention techniques. To help the model locate various lesions in complex fundus images more precisely, cross-attention is employed to efficiently integrate important vascular information, while self-attention is utilized to identify long-distance dependence links among lesions. Attention Gates (AG) in skip connections improve lesion localization, while CBAM modules in both the encoder and decoder layers are integrated into our CBAM-AG-UNet for adaptive feature improvement. By tackling these issues, our suggested approach guarantees more accurate and trustworthy microaneurysm identification, improving the segmentation process’s efficiency and resilience. This approach ensures improved detection of tiny MAs, making sure that even faint microaneurysms are accurately segmented. As a result, our model achieves a Dice coefficient of 0.865, an IoU of 0.758, an inference time of 0.3392 s, memory usage was 17.11 MB and an AUC of 0.996, significantly outperforming state-of-the-art segmentation models (as shown in Table 5).

**Table 5:** Comparison of our proposed CBAM-AG-UNet with the state-of-the-art approaches on the IDRiD dataset

Author	Method/model	Dice	IoU	AUC
Wang et al. [35]	MCA-UNet	0.3850	-	0.9912
Jiang et al. [36]	Galance Seg	0.3944	-	-
Xu et al. [37]	MSAG	0.4920	-	-
Zhang et al. [38]	TC-UNet	0.5696	0.6968	0.9881
Xu et al. [39]	Attention fusion transformer encoder (AFTE)	-	0.2834	-
He et al. [40]	PMC Net	-	-	0.4694
Song guo et al. [41]	end-to-end unified framework L-Seg	-	-	0.4627
Proposed method	CBAM-AG-UNet Framework	0.865	0.758	0.996

A ‘-’ (dash) is used to indicate that some of the items in Table 5. employ different evaluation measures. While some studies evaluate performance using metrics such as Area Under the Curve (AUC), Dice coefficient, and Intersection over Union (IoU), others may report just one or two of these metrics. As a result, the missing numbers in these columns represent the various performance metrics used in those studies rather than representing partial results. In subsequent iterations of this study, we intend to connect the findings with widely used metrics such as IoU and Dice, acknowledging the necessity of standardized evaluation criteria to enable comparisons.

## Conclusion

In this study, we proposed a unique UNet architecture for microaneurysm segmentation in which the encoder and decoder use Convolutional Block Attention Modules (CBAM) and the skip connections use Attention Gates (AG). The encoder’s incorporation of CBAM enhances feature extraction and ensures that the most relevant and instructional features are extracted by leveraging channel and spatial attention processes. The Attention Gates in the skip connections further refine this information by emphasizing significant features, which makes it easier to allow the decoder to get critical data. Adding CBAM inside the decoder also refines upsampled features while retaining high-quality visualization and spatial information. The comprehensive technique known as Three-fold Attention significantly raises segmentation accuracy. Several experiments demonstrated that our approach outperformed earlier ones, indicating its potential for improved clinical microaneurysm detection and examination. According to our findings, medical image segmentation can be enhanced by attention mechanisms, which pave the way for further research and development in this area.

## Data Availability

Availability of data and materials - IDRiD Dataset: https://ieee-dataport.org/open-access/indian-diabetic-retinopathy-image-dataset-idrid.

## Footnotes

1 https://ieee-dataport.org/open-access/indian-diabetic-retinopathy-image-dataset-idrid.

## Abbreviations

DR: Diabetic retinopathy
NPDR: Non-proliferative diabetic retinopathy
PDR: Proliferative diabetic retinopathy
MA: Microaneurysms
CBAM: Convolutional block attention module
CAM: Channel attention module
SAM: Spatial attention module
AG: Attention gate
AUC: Area under the curve
FC: Fully connected
ROC: Receiver operating characteristic
GPU: Graphical processing unit
CLAHE: Contrast limited adaptive histogram equalization
DSC: Dice similarity coefficient
IOU: Intersection over union

## References

[1] Ahmed NGA, Hamza MF, Hassan SN. Knowledge, practice and attitude of diabetic patients regarding the prevention of diabetic retinopathy. J Surv Fisher Sci. 2023;10(3S):3896–908.

[2] Bai Y, Zhang X, Wang C, Gu H, Zhao M, Shi F. Microaneurysms detection in retinal fundus images based on shape constraint with region-context features. Biomed Signal Process Control. 2023;85:104903.

[3] Yau JW, Rogers SL, Kawasaki R, et al. Global prevalence and major risk factors of diabetic retinopathy. Diabetes Care. 2012;35(3):556–64. 22301125 10.2337/dc11-1909 PMC3322721

[4] Fleming AD, Philip S, Goatman KA, Olson JA, Sharp PF. Automated microaneurysm detection using local contrast normalization and local vessel detection, IEEE Trans Med Imag. 2006;25(9):1223–1232. 10.1109/tmi.2006.879953 16967807

[5] Quellec G, Lamard M, Josselin PM, Cazuguel G, Cochener B, Roux C. Optimal wavelet transform for the detection of microaneurysms in retina photographs, IEEE Trans. Med. Imag. Sep. 2008;27(9):1230–1241. 10.1109/TMI.2008.920619 PMC2567825 18779064

[6] Chudzik P, Majumdar S, Caliva F, Al-Diri B, Hunter A. Microa aneurysm detection using fully convolutional neural networks. Comput Methods Programs Biomed. May 2018;158:185–92. 10.1016/j.cmpb.2018.02.016 29544784

[7] Zhang X, et al. T-net: hierarchical pyramid network for microaneurysm detection in retinal fundus image. IEEE Trans Instrum Meas. 2023;72:1–13. 10.1109/TIM.2023.3286003.37323850

[8] Zhang X, Kuang Y, Yao J. Detection of microaneurysms in color fundus images based on local fourier transform. Biomed Signal Process Control. 2022;76(Jul):Art103648.

[9] Soares I, Castelo-Branco M, Pinheiro A. Jan., Microaneurysms detection in retinal images using a multi-scale approach. Biomed Signal Process Control, 79, 2023, Art. 104184.

[10] Zhang X, Ma Y, Gong Q, Yao J. Aug., Automatic detection of microaneurysms in fundus images based on multiple preprocessing fusion to extract features. Biomed Signal Process Control, 85, 2023, Art. 104879.

[11] Gao W, Fan B, Fang Y, Shan M, Song N. Detection and location of microaneurysms in fundus images based on improved YOLOv4 with IFCM, IET Image Process. Sep. 2023;17(11):3349–3357.

[12] Wang Z, Li X, Yao M, Li J, Jiang Q, Yan B. A new detection model of microaneurysms based on improved FC-DenseNet. Sci Rep. Jan. 2022;12(1):950. 10.1038/s41598-021-04750-2 PMC8770497 35046432

[13] Tan JH et al. Dec., Automated segmentation of exudates, hemorrhages, microaneurysms using single convolutional neural network, Inf. Sci. 2017;420:66–76.

[14] Ronneberger O, Fischer P, Brox T. U-net: Convolutional networks for biomedical image segmentation. 2015;arXiv:1505.04597.

[15] Zhang Z, Liu Q, Wang Y. Road extraction by deep residual U net. IEEE Geosci Remote Sens Lett. 2018;15:749–53.

[16] Kou C, Li W, Liang W, Yu Z, Hao J. Microaneurysms segmentation with a U-Net based on recurrent residual convolutional neural network, J. Med. Imag. Jun. 2019;6(2):1. 10.1117/1.JMI.6.2.025008 PMC6582229 31259200

[17] Kou C, Li W, Yu Z, Yuan L. An enhanced residual U-Net for microaneurysms and exudates segmentation in fundus images. IEEE Access. 2020;8:185514–25.

[18] Qomariah D, Tjandrasa H, Fatichah C. Segmentation of microa neurysms for early detection of diabetic retinopathy using MResUNet, Int. J. Intell. Eng. Syst. Jun. 2021;14(3):359–373.

[19] Xu Y, Zhou Z, Li X, Zhang N, Zhang M, Wei P. FFU-net: Feature fusion U-net for lesion segmentation of diabetic retinopathy, BioMed Res. Int. Jan. 2021;2021:1–12. 10.1155/2021/6644071 PMC7801055 33490274

[20] Bhargav PR, Puhan NB. Novel contra harmonic cor relative attention loss for microaneurysm segmentation in fundus images, IEEE Sensors Lett. Jul. 2023;7(7):1–4. 10.1109/LSENS.2023.3290597

[21] Chen J, Chen C, Huang W, Zhang J, Debattista K, Han J. Dynamic contrastive learning guided by class confidence and confusion degree for medical image segmentation. Pattern Recogn. 2024;145:109881.

[22] Behera SS, Puhan NB. High boost 3-D attention network for cross-spectral periocular recognition, IEEE Sensors Lett. Sep. 2022;6(9):1–4.

[23] Zhang Z, Liu Q, Wang Y. Road extraction by deep residual U-Net. IEEE Geosci Remote Sens Lett. May 2018;15(5):749–53.

[24] Oktay O et al. Attention U-Net: learning where to look for the pancreas, 2018, arXiv:1804.03999.

[25] Dosovitskiy A et al. An image is worth 16×16 words: Transformers for image recognition at scale, 2020, arXiv:2010.11929.

[26] Guo D, Terzopoulos D. A transformer-based network for anisotropic 3D medical image segmentation, in Proc. 25th Int. Conf. Pattern Recognit. (ICPR), Jan. 2021:8857–8861.

[27] Chen C-F, Fan Q, Panda R. CrossViT: Cross-attention multi-scale vision transformer for image classification, in 2021 IEEE/CVF International Conference on Computer Vision (ICCV). 2021:347–356.

[28] Ding M, Qu A, Zhong H, Liang H. A transformer-based network for pathology image classification, in: 2021 IEEE International Conference on Bioinformatics and Biomedicine (BIBM). 2021:2028–2034.

[29] Chen J, Lu Y, Yu Q, Luo X, Adeli E, Wang Y, Lu L, Alan LY. Yuyin Zhou, Transunet: Transformers Make Strong Encoders for Medical Image Segmentation. 2021 arXiv preprint arXiv:2102.04306.

[30] Cao H, Wang Y, Chen J, Jiang D, Zhang X, Qi T, Wang M. Swin-unet: Unet-like Pure Transformer for Medical Image Segmentation. 2021 arXiv preprint arXiv:2105.05537.

[31] Li Y, Wang Z, Yin L, Zhu Z, Qi G, Liu Y. X-Net: a dual Encoding-Decoding method in medical image segmentation. The Visual Computer. 2021:1–11.

[32] Chen J, Zhang J, Debattista K, Han J. Semi-supervised unpaired medical image segmentation through task-affinity consistency. IEEE Trans Med Imaging. 2022;42(3):594–605. 10.1109/TMI.2022.3213372 36219664

[33] Sonali SS, Singh AK, et al. An approach for de-noising and contrast enhancement of retinal fundus image using CLAHE. Opt Laser Technol. 2019;110:87–98.

[34] Woo S, Park J, Lee J, Kweon I. CBAM: Convolutional block attention module. arXiv 2018;1:3–19. 10.1007/978-3-030-01234-2

[35] Wang H, Cao P, Yang J, Zaiane O. MCA-UNet: multi-scale cross co-attentional U-Net for automatic medical image segmentation. Health Inform Sci Syst. 2023;11(1):10. 10.1007/s13755-022-00209-4 PMC9884736 36721640

[36] Jiang, H., Gao, M., Liu, Z., Tang, C., Zhang, X., Jiang, S., … Liu, J. GlanceSeg:Real-time microaneurysm lesion segmentation with gaze-map-guided foundation model for early detection of diabetic retinopathy. IEEE Journal of Biomedical and Health Informatics. 2024. 10.1109/JBHI.2024.3377592 38483801

[37] Xu C, He S, Li H. An attentional mechanism model for segmenting multiple lesion regions in the diabetic retina. Sci Rep. 2024;14(1):21354. 39266650 10.1038/s41598-024-72481-1 PMC11392929

[38] Zhang Z, Sun G, Zheng K, Yang JK, Zhu XR, Li Y. TC-Net: A joint learning framework based on CNN and vision transformer for multi-lesion medical image segmentation. Comput Biol Med. 2023;161:106967. 37220707 10.1016/j.compbiomed.2023.106967

[39] Xu C, Guo X, Yang G, Cui Y, Su L, Dong H, Che S. Prior-guided attention fusion transformer for multi-lesion segmentation of diabetic retinopathy. Sci Rep. 2024;14(1):20892. 39245695 10.1038/s41598-024-71650-6 PMC11381548

[40] He A, Wang K, Li T, Bo W, Kang H, Fu H. Progressive multiscale consistent network for multiclass fundus lesion segmentation. IEEE Trans Med Imaging. 2022;41(11):3146–57. 35613070 10.1109/TMI.2022.3177803

[41] Guo S, Li T, Kang H, Li N, Zhang Y, Wang K. L-Seg: an end-to-end unified framework for multi-lesion segmentation of fundus images. Neurocomputing. 2019;349:52–63.

